# Epidemiology and outcomes of trauma patients at The Indus Hospital, Karachi, Pakistan, 2017 – 2018

**DOI:** 10.12669/pjms.36.ICON-Suppl.1717

**Published:** 2020-01

**Authors:** Saima Salman, Syed Ghazanfar Saleem, Quratulain Shaikh, Anna Q Yaffee

**Affiliations:** 1 Saima Salman, Consultant, Emergency Department, The Indus Hospital Research Center, The Indus Hospital, Karachi, Pakistan; 2 Syed Ghazanfar Saleem, Consultant, Emergency Department, The Indus Hospital Research Center, The Indus Hospital, Karachi, Pakistan; 3 Quratulain Shaikh, Epidemiologist, The Indus Hospital Research Center, The Indus Hospital, Karachi, Pakistan; 4 Anna Q Yaffee, Consultant EM, Grady Memorial Hospital, Emory University, Atlanta, USA

**Keywords:** Injury, Injury Severity Score (ISS), Low-and Middle-Income Countries (LMIC), Trauma

## Abstract

**Objective:**

Structured trauma care has proven to improve patient outcomes, and this is more relevant in the low- and middle-income countries (LMICs). The objective of this study was to determine the distribution, etiology, severity and outcomes of trauma patients at the Indus Hospital.

**Methods:**

All adult poly-trauma patients presenting to The Indus Hospital from July 2017 to June 2018 were included in this retrospective review. Data was extracted on etiology of trauma, severity of injury, investigations and final disposition of patients.

**Results:**

Of 972 trauma patients presenting to TIH Emergency Department, 663 (68.2%) were males with a mean age of 36 (17.4) years. Road traffic accidents (RTAs) led to trauma in 766 patients (78.8%), followed by 121 falls (12.7%). Injury Severity score (ISS) was calculated upon arrival and 528 (54.3%) were found to be critically injured. Median length of stay was 60 (24-720) minutes while none utilized pre-hospital Emergency Medical services.

**Conclusion:**

Most trauma patients were males suffering from RTA. Nearly half of the patients were critically injured on arrival. EMS is not utilized by trauma patients. There were gaps identified in the diagnosis and treatment of trauma.

## INTRODUCTION

Globally, trauma represents an overwhelming burden of morbidity and mortality where 16,000 people die each day from injuries; Ninety percent occur low- and middle-income countries (LMICs).^1^,^2^ For each death, three hundred times as many patients reach the ED, indicating large ED volumes of injured patients.^3^ In addition, LMICs from Southeast Asia and western Pacific regions have the highest number of worldwide deaths due to injuries.^4^ Lack of health care infrastructure, safety promotion and proper legislature, possibly due to political instability in these countries, has resulted in low budget allocation to injury prevention and trauma care.

Pakistan is the sixth most populous country in the world with the 2017 National Census reporting more than 200 million people. The Pakistan Bureau of Statistics shows that road traffic accidents during 2008-2018 led to an average of 4907-5948 deaths and 11,037-14,489 injuries per year.^5^ National surveys conducted in Pakistan over the years have identified trauma as one of the leading causes of morbidity and mortality, predominantly affecting young males.^6^ This leads to loss of income for both the patient and their caregivers and increased out-of-pocket healthcare expenses.^7^

Previous studies have identified major gaps in quality emergency and trauma care in Pakistan.^8^,^9^ These include lack of public awareness regarding road safety^10^, lack of robust pre-hospital emergency medical services (EMS) across the country and inconsistent availability of basic resources including trained personnel at primary and district health care facilities.^11^,^12^

The Indus Hospital, Korangi (TIH) is a private sector, free of cost hospital in Karachi with approximately 190,000 Emergency Department (ED) patient visits annually. TIH ED is a limited resource center but in spite of this, has recently established training programs to focus on more robust emergency education and practice, prioritizing trauma as a preventable cause of death. Being one of seven hospitals recognized across Pakistan to train fellows in Emergency Medicine, the aim is to streamline trauma patient management in accordance with current recommendations and guidelines to improve outcomes.

Through characterizing the epidemiology of trauma patients at TIH, we hope to understand the current burden of injury presenting to our ED, the demographic distribution of trauma patients, Injury Severity Score (ISS)^13^ upon arrival and patient outcomes. We aimed to identify gaps in the management of trauma patients at TIH to guide the establishment of a team approach to standardize trauma management. This will lead to planning of future ED based interventions and identify further areas of capacity building through training and teaching.

## METHODS

A retrospective chart review of all poly-trauma patients older than 14 years who presented to The Indus Hospital, Emergency Department (July 1, 2017 – June 30, 2018) was performed. Study was exempted from IRB approval under number (IRD_IRB_2018_12_002). Data was extracted through the help of Health Management Information Systems (HMIS) and entered on pre-designed questionnaire to recording the demographics, mechanism of injury and ISS score calculated upon arrival and investigations performed in the Emergency Department after clinical assessment.^11^ The Injury Severity Score (ISS) is an anatomical scoring system that provides an overall score for patients with multiple injuries. Each injury is assigned an Abbreviated Injury Scale (AIS) score and is allocated to one of six body regions (Head, Face, Chest, Abdomen, Extremities (including Pelvis), External). Only the highest AIS score in each body region is used. The three most severely injured body regions have their score squared and added together to produce the ISS score.

### Statistical analysis

Data analysis was done on SPSS version 24. Descriptive statistics were reported as mean with standard deviation or median with interquartile range for continuous data and frequency with percentage for categorical data. Chi-square or Fischer’s Exact test was used as appropriate to test the difference between categorical variables. A p-value less than 0.05 was considered significant.

## RESULTS

Medical records of 972 trauma patients presenting to TIH ED were reviewed revealing 663 (68.2%) males. The median length of stay was 60 (24-720) minutes with no provision of pre-hospital Emergency Medical Service (EMS) in any case. Road traffic accidents were the most common cause of trauma, seen in 766 patients (78.8%) followed by 121 patients with falls (12.7%). The musculoskeletal system was involved in most patients (n=740; 76.1%) followed by head and neck injuries in 491 (50.5%) patients and chest trauma in 133 (13.7%) patients (Table-I).

**Table I:** Characteristics of Trauma Patients (N=972).

	N (972)	(%)
*Age (years)* (Mean, SD)	36	17.4
Male	663	68.2
*Length of Stay (minutes)* (Median, Range)	60	24-720
Pre-hospital Emergency Medical Services	0	0
*Injury Type*		
Road Traffic Accident (RTA)	766	78.8
Industrial Injury	12	1.2
Assault	47	4.8
Burn	26	2.6
Fall	121	12.4
*Region of Body Involved*		
Head & Neck	491	50.5
Chest	133	13.7
Abdomen	31	3.2
Musculoskeletal	740	76.1
Facial Trauma	86	8.8
*Injury Severity Score (ISS)*		
Minor (1-8)	0	0
Moderate (9-15)	386	39.7
Serious (16-24)	32	3.2
Severe (25-49)	17	1.7
Critical (50-74)	528	54.3
Maximum (75)	9	0.9
*Disposition*		
Admit	23	2.4
Transfer	542	55.8
Left Against Medical Advice (LAMA)	42	4.3
Discharged	356	36.6
Expired	9	0.9

Injury Severity Score (ISS) was calculated upon arrival and 528 (54.3%) were found to be critically injured (ISS =50-74). Out of these critically injured patients only 23 patients (4%) were admitted, of whom two died and 21 were discharged home. Transfer of 505 (96%) patients were required due to limitations in infrastructure and lack of required specialties at TIH namely, neurosurgery and vascular surgery. All 356 discharged patients (100%) were moderately injured with ISS 9-15. Conversely, all maximally injured nine patients with ISS 75 died. Only 3.4% of maximally injured patients received a Focused Assessment Sonography in Trauma (FAST) ultrasound and none received further helical imaging of chest or abdomen. A chest radiograph was performed in only 254 patients (26.1%). Intravenous fluids were given to 90% but only 3.4% received blood transfusion. Patients with critical ISS had fewer investigations and interventions than patients with lower or maximum ISS. (Table-II & III). Industrial trauma (p=0.01) and falls (p=0.007) were more common in men whereas burn victims were mostly women (p=0.0001) (Table-IV).

**Table II:** Distribution of ISS with Disposition.

	Moderate (386)	Serious (32)	Severe (17)	Critical (528)	Maximum (9)
Admit (23)	0	0	0	23 (4%)	0
Transfer (542)	0	23 (72%)	14 (82%)	505 (97%)	0
LAMA (42)	30 (71%)	9 (28%)	3 (18%)	0	0
Discharged (356)	356 (100%)	0	0	0	0
Expired (9)	0	0	0	0	9 (100%)

**Table III:** Distribution of ISS with Intervention.

	Moderate (386)	Serious (32)	Severe (17)	Critical (528)	Maximum (9)
C spine stabilization (84)	10 (3%)	17 (53%)	17 (100%)	31 (6%)	9 (100%)
ETT (33)	0	0	8 (47%)	16 (3%)	9 (100%)
Chest tube thoracostomy (26)	0	3 (9%)	8 (47%)	10 (2%)	5 (56%)
Pelvic stabilization (30)	0	2 (6%)	7 (41%)	17 (3%)	4 (44%)
Laceration repair (77)	55 (14%)	2 (6%)	8 (47%)	7 (1%)	5 (56%)
Blood Tx (23)	0	1 (3%)	4 (24%)	9 (3%)	9 (100%)
I/V fluids (875)	289 (75%)	32 (100%)	17 (100%)	528 (100%)	9 (100%)

**Table IV:** Distribution of Injuries by Gender.

	Female, N (%)	Male, N (%)	p value
*Road Traffic Accident*			
Yes	247 (80)	519 (78)	0.5
No	62 (20)	144 (22)	
*Industrial Trauma*			
Yes	0 (0)	12 (1.8)	0.01
No	309 (100)	651 (98)	
*Falls*			
Yes	26 (8.4)	96 (14.4)	0.007
No	283 (91.5)	568 (85.6)	
*Assault*			
Yes	18 (5.8)	29 (4.3)	0.3
No	291 (94)	634 (95.6)	
*Burns*			
Yes	18 (5.8)	8 (1.2)	<0.0001
No	291 (94)	655 (99)	

There was incomplete documentation from all specialties managing trauma patients and a lack of coordinated patient care in accordance to recommended guidelines, was observed. This, as well as delayed patient throughout time in ED, and insufficient ordering of point-of-care investigations as well as inadequate safe patient referral and disposition were identified through the available documentation of patient records.

## DISCUSSION

In this description of trauma epidemiology presenting to the Indus Hospital in Karachi, Pakistan, the majority of our patients were male, and most commonly presented with road traffic accident or fall. This is consistent with prior local literature suggesting victims of trauma in the region are often male and due to blunt force trauma (road traffic accidents, falls, assault). The epidemiology of trauma patients presenting at TIH is therefore broadly reflective of trauma seen across Pakistan.^6^,^14^,^15^ The high incidence of road traffic accidents and falls identified in our study could be due to a lack of awareness about road safety and use of protective equipment on construction sites. Hence, injuries to the musculoskeletal system were most commonly observed. Burns were common in women, potentially due to use of fire for cooking in the home or possibly due to domestic abuse reported in patient records and consistent with reports elsewhere.^16^-^18^ Societal determinants like poverty and lack of education may be the underlying root cause of trauma in our patient population. In addition, lack of legislature and policies, and their enforcement at a national level on issues such as building safety codes (e.g. use of personal protective equipment), and road safety (e.g. speed control on roads with seat belt, child restraint and helmet use) lead to additional trauma burden.^6^ The recent incorporation of the Amal Act into the constitution is a step forward in ensuring safety of patients in Emergency Departments, but there is still a long way to go.^19^

Although the majority of our patients were critically injured based on ISS, it was found that the use of investigations was not equitable. The critically injured patients tended to receive fewer investigations and interventions, compared with the maximally or less injured patients. Standard trauma care are, at minimum, is expected to include an ultrasound FAST and chest radiograph on critically injured poly-trauma patients. These findings offer an opportunity for improvement in critical trauma patient care and would benefit from a standardized proven approach to trauma, such as establishment of trauma teams.

The lack of organized EMS presents an added challenge for our patients.^10^ A higher ISS on initial presentation may be associated with the fact that none of our patients were brought to hospital by EMS, which enables earlier initiation of emergency care. The first hour of trauma (‘the golden hour’) is the most effective in impacting morbidity and mortality. Whilst the majority of our patients were discharged from the ED after a median 60 (24-720) minutes, over 90% of our critically injured patients required transfer. For 188 critically injured patients this was due to lack of beds at TIH and for 542 the absence of required subspecialty services, namely neurosurgery and cardiothoracic surgery, delayed their access to definitive care. The EMS care in Karachi is shared by non-governmental organizations with limited data on their impact and performance evaluation.^14^,^18^ A structured, coordinated EMS system for Karachi could alleviate some of these concerns, allowing patients to be stabilized in route and taken first to hospitals with the appropriate level of service for their injury. The need for well-developed EMS has been highlighted in various national studies with emphasis on safe referrals between institutes.^20^ In addition, as TIH expands, we plan to offer additional specialized services so these patients to be cared for in-house.

The opportunities for improvement are vast. Collaborative capacity building for ED and surgical personnel through structured trauma training, efficient utilization of available resources, and better documentation from all stakeholders is important to improve outcomes for patient care. In addition, a centralized city wide approach to improve hospital liaison and efficient, well trained EMS services can ensure better organized and continuous trauma care.

## CONCLUSION

Trauma was a common reason for presentation to ED at TIH. Most patients were male and etiology in majority was RTA or fall. Gaps were identified in management of trauma patients. Most suffered from musculoskeletal system injuries and had critical ISS at presentation. None of the patients received pre-hospital EMS in our study.

### Recommendations

Understanding trauma patient epidemiology and management at TIH has provided an insight into the current services and opened potential options of better coordination and capacity building amongst key stakeholders. Through identifying the gaps, it is our hope that ED based interventions such as organized trauma team approach for patient management, capacity building through formal teaching and intelligent resource utilization will result in effective and well-coordinated patient disposition. The opportunity for collaboration across the city and then the country through improved trauma referral network will have a positive impact on our trauma patient outcomes.

### Author`s Contribution

**SS, GS, and AY:** Conceptualization of project and research guidance.

**SS and GS:** Manuscript writing and literature search, takes responsibility for integrity of research.

**QS:** Data analysis and results write up.

**AY:** Data analysis, proof reading and editing.

## References

[1] Ogo CN, Kolawole HF, Eyesan SU, Obalum DC (2013). Challenges in trauma management in a developing economy. OA Orthopaedics.

[2] Joshipura M, Mock C, Goosen J, Peden M (2004). Essential Trauma Care:strengthening trauma systems round the world. Injury.

[3] (2002). Injuries WHO, Department VP, Organization WH, Injuries WHODo, Prevention V. The injury chart book:A graphical overview of the global burden of injuries:World Health Organization.

[4] Razzak JA, Sasser SM, Kellermann AL (2005). Injury prevention and other international public health initiatives. Emerg Med Clin North Am.

[5] Statistics PBo Traffic Accidents (Annual).

[6] Hyder AA, Razzak JA (2013). The challenges of injuries and trauma in Pakistan:an opportunity for concerted action. Public Health.

[7] Economics T Pakistan GDP per capita.

[8] Government of Sindh. Updated 2011.

[9] Islamabad Pes (2011). Economic Advisor's Wing, Finance Division, Government of Pakistan.

[10] Farooq U, Bhatti JA, Siddiq M, Majeed M, Malik N, Razzak JA (2011). Road traffic injuries in Rawalpindi city, Pakistan. East Mediterr Health J.

[11] Razzak JA, Hyder AA, Akhtar T, Khan M, Khan UR (2008). Assessing emergency medical care in low income countries:a pilot study from Pakistan. BMC Emerg Med.

[12] Razzak JA, Baqir SM, Khan UR, Heller D, Bhatti J, Hyder AA (2015). Emergency and trauma care in Pakistan:A cross-sectional study of healthcare levels. Emerg Med J.

[13] Baker SP, O'Neill B (1976). The injury severity score:an update. J Trauma.

[14] Waseem H, Naseer R, Razzak JA (2011). Establishing a successful pre-hospital emergency service in a developing country:experience from Rescue 1122 service in Pakistan. Emerg Med J.

[15] Jafar TH, Haaland BA, Rahman A, Razzak JA, Bilger M, Naghavi M (2013). Non-communicable diseases and injuries in Pakistan:strategic priorities. Lancet.

[16] Ghaffar A, Hyder AA, Masud TI (2004). The burden of road traffic injuries in developing countries:the 1st national injury survey of Pakistan. Public Health.

[17] Statistics PFBo (2011). Pakistan Demographic survey – 2005.

[18] Karmaliani R, Irfan F, Bann CM, McClure EM, Moss N, Pasha O (2008). Domestic violence prior to and during pregnancy among Pakistani women. Acta Obstet Gynecol Scand.

[19] (2019). Provincial Assembly of Sindh Notification Karachi.

[20] Khan S, Zafar H, Zafar SN, Haroon N (2014). Inter-facility transfer of surgical emergencies in a developing country:effects on management and surgical outcomes. World J Surg.

